# Delayed Pneumothorax Following Bougie-Assisted Nasal Intubation

**DOI:** 10.7759/cureus.56225

**Published:** 2024-03-15

**Authors:** Lisa Bethea

**Affiliations:** 1 Anesthesiology, Moffitt Cancer Center, Tampa, USA

**Keywords:** bougie and pneumothorax, pneumothorax and intubation, intubation complications, nasal intubation, traumatic pneumothorax, endotracheal intubation, gum elastic bougie

## Abstract

A 45-year-old male with tongue cancer and obstructive sleep apnea presented for glossectomy and right neck dissection. He underwent a difficult nasal intubation assisted by a bougie and Glidescope. After an otherwise uneventful procedure, the patient was extubated and taken to recovery. Several hours later, he developed increased respirations and decreased oxygen (O_2_) saturation with decreased air movement on the right side of his chest. A chest X-ray confirmed a right pneumothorax. A chest tube was placed with immediate improvement of O_2_ saturation and breathing. Pneumothorax was presumably due to trauma from intubation. Although pneumothorax is a potential complication of intubation, it is more likely to occur shortly following intubation instead of hours later. The mechanism is often unknown. Providers must monitor patients throughout the perioperative period for any potential respiratory concerns, especially following a difficult intubation. This will ensure prompt diagnosis and management of any complications and provide an optimal outcome for the patient.

## Introduction

General anesthesia is not without adverse events, especially during intubation. This is a critical time in patient care when precautions are taken to ensure an uneventful intubation process. Intubation can occur via the oral or nasal route and can be carried out via direct laryngoscopy, Glidescope, or a fiberoptic bronchoscope [1]. Additional tools that could be utilized include a gum elastic bougie, intubating stylet, or lightwands [1]. These tools can make intubation more seamless; however, complications can still occur. A rare but potentially life-threatening complication is pneumothorax. It is most often associated with difficult intubation, the use of a double-lumen tube, or the placement of a central line [2]. This is a case report of delayed pneumothorax after bougie-assisted nasal intubation in a patient undergoing a head and neck procedure. Although an uncommon occurrence, bougie has been linked to pneumothorax following intubation, particularly in difficult situations [2-5].

## Case presentation

**Figure 1:**
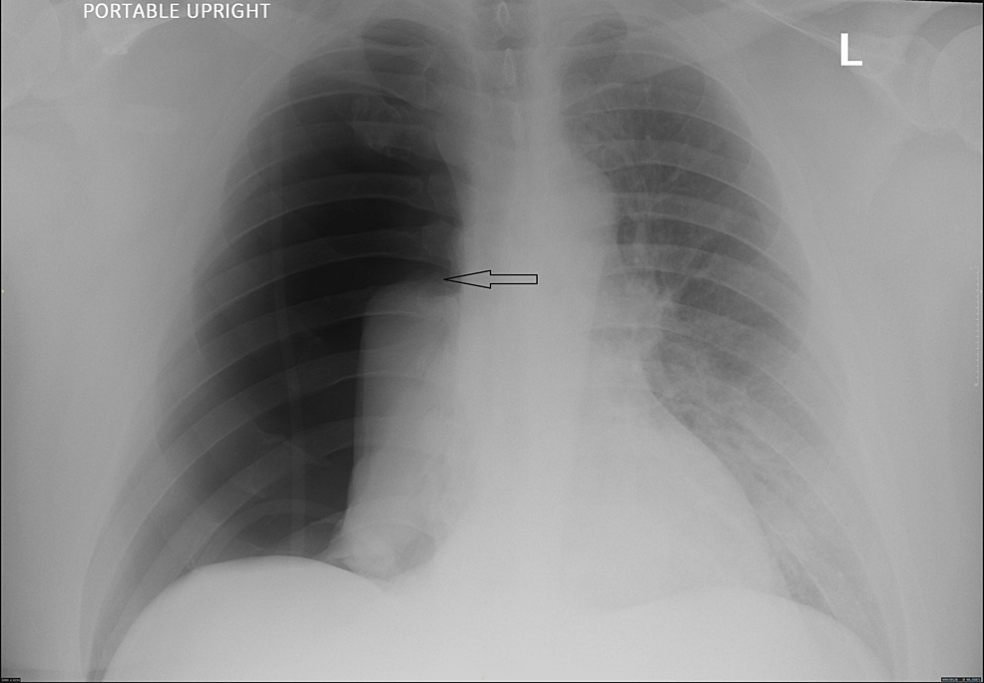
Large right-sided pneumothorax

**Figure 2:**
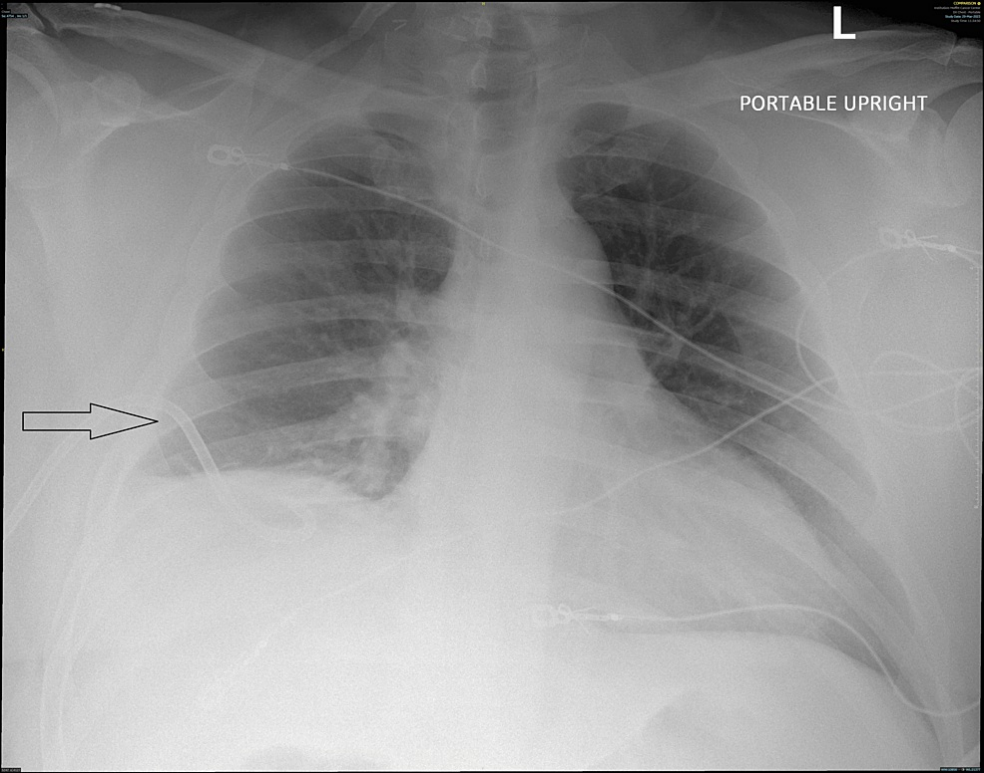
Re-inflated right lung after tube thoracostomy

A 45-year-old male with a past medical history of obstructive sleep apnea (OSA), treated with continuous positive airway pressure (CPAP), and tongue cancer presented for glossectomy and neck dissection under general anesthesia. His weight was 126 kg with a BMI of 39. His presenting vital signs were within normal limits, and his labs were unremarkable. Given his otherwise negative medical history and the nature of the procedure, no additional studies were required, including electrocardiogram (EKG) and chest x-ray (CXR). He did have a CT scan of the neck for surgical planning that was negative. On exam, he had a Mallampati III airway with a thick, short neck. Neck mobility was not limited. The plan was for nasal intubation using Glidescope and Magill forceps for assistance if needed. The patient was taken to the operating room and moved to the operating table. Standard ASA monitors were applied, and the patient was preoxygenated with 100% O_2_ via a bag mask. He was induced with IV propofol, lidocaine, fentanyl, and rocuronium. Both nares were assessed, and the left was chosen for intubation. A pre-softened, lubricated 7.5 mm I.D. endotracheal tube (ETT) was placed in the left nares with some resistance. The ETT was removed, and the nares dilated with a nasal airway. Again, the ETT was placed in the left nares but with no resistance. The Glidescope was then used to visualize the ETT in the pharynx and assist with placement through the vocal cords. The ETT was unable to be maneuvered directly through the cords. Magill forceps were used to grab the ETT and assist with placement through the cords. This required some additional twisting of the ETT as well as jaw thrust and cricoid pressure to help guide the ETT through the cords. ETT cuff was inflated, and bilateral breath sounds were noted as well as positive end-tidal carbon dioxide (ETCO_2_). Shortly thereafter, a leak was noted within the circuit. The patient and anesthesia circuit system were assessed. The ETT balloon cuff was noted to be deflated and was not maintaining air despite adding air multiple times. It was determined that the ETT cuff must have been inadvertently punctured during tube placement. Because initial placement was somewhat difficult, the decision was made to exchange the ETT over a bougie. The bougie was lubricated and placed through the original ETT without difficulty, and the ETT was removed. A new ETT was guided over the bougie and into the airway under Glidescope guidance. The bougie was removed. Bilateral breath sounds were confirmed along with positive ETCO_2_. The ETT was secured in place. Following intubation, a second 18 gauge IV was placed as well as a left radial arterial line. The surgeon proceeded with a glossectomy and right neck dissection, which did not involve the airway or thorax. The patient was maintained on Sevoflurane anesthesia with a 50% FiO_2_ oxygen/air mixture. The vital signs, including oxygen (O_2_) saturation, remained stable, airway pressure was <40 throughout the procedure, and there were no signs of respiratory concerns. After an otherwise uneventful four-hour procedure, the patient was extubated to room air. His initial O_2_ saturation was in the high 70s to low 80s. Bag mask ventilation improved the O_2_ level to the 90s. The patient was placed on O_2_ 2L via a nasal cannula and taken to the post-anesthesia care unit (PACU). While in the PACU, his O_2_ saturation was in the low to mid-90s. He was given a nebulizer treatment and then placed on CPAP secondary to his history of sleep apnea. The patient reported not being able to tolerate the CPAP and was switched to O_2_ at 2L via a nasal cannula (NC). His O_2_ saturation was maintained at 93-98% with respirations of 20. The patient continued to recover without incident and was transferred to the inpatient ward. However, several hours post-procedure, he was noted to be dyspneic with increased O_2_ requirements of 5 to 10L NC and O_2_ saturation of 90%. Again, CPAP was attempted without success. The patient also reported pleuritic chest pain, which worsened after CPAP was placed. On exam, he had absent breath sounds on the right and crackles in the left lower lung. A CXR was obtained showing a large right-sided pneumothorax (Figure 1). The patient was immediately transferred to the intensive care unit (ICU) for tube thoracostomy. A right-sided chest tube was placed without difficulty. Immediately, the patient had improvement in his O_2_ saturation and breathing. A repeat CXR showed a resolution of the pneumothorax (Figure 2). The patient was monitored overnight in the ICU. His O_2_ requirement decreased, and he was maintaining 100% O_2_ saturation on room air by the next morning. The chest tube remained in place for two days with a trial of CPAP while the chest tube was in place to ensure no recurrence of pneumothorax. The chest tube was removed without incident. The patient continued to do well with no further respiratory issues. He was discharged home the following day. He had no subsequent issues post-discharge. About three months following the procedure, a CT chest was obtained. There were no abnormalities present.

## Discussion

Nasal intubation is an alternative method of securing the airway for general anesthesia. It is often used for various maxillofacial procedures involving the oral cavity but can also be used in patients with oral abnormalities (masses in the mouth or decreased mouth opening) [6,7]. Nasal intubation should be avoided in patients with basal skull fractures or midface instabilities, coagulopathies, epiglottitis, nasal lesions, or recent nasal surgery [6,7]. It is important to be aware of the patient's anatomy to ensure proper placement as well as avoid any potential complications. Various methods have been used to facilitate nasal intubation. The most commonly used method involves blindly placing the ETT through the nares and into the oral cavity, and then via direct laryngoscopy, using the Magill forceps to navigate the tube through the vocal cords [6,7]. Although this method is often successful, problems can still arise. Advancing the ETT through the nasal passage and into the airway can prove difficult. It may require twisting, turning, and forced manipulation of the ETT causing damage to surrounding structures as well as the ETT itself [6,7]. Other methods for nasotracheal intubation include stylet-assisted intubation, light wand technique, fiber-optic intubation, and bougie-facilitated intubation [6,7]. These alternative methods can be more advantageous when placement of the nasotracheal tube is difficult. Abrons et al. described a series of cases that successfully utilized the bougie to secure the airway nasally [8]. This report suggests that using the bougie for nasal intubation assistance can not only cause less bleeding, but it can also reduce damage to surrounding structures as well as the ETT [8].

Airway exchange catheters like the bougie are more readily available than many other airway-assist devices. It is often kept in the anesthesia cart or with difficult airway kits. A bougie is a flexible stylet-type device about 60 cm long with a 15 French diameter and angled tip [1,9]. It is primarily used to help facilitate the placement of an ETT, especially in difficult airways. It can also be used to assist with a surgical airway or to exchange out an ETT, although tube exchangers are preferable [9]. It is inserted directly into the trachea, and then the ETT can be guided over it. As the bougie is placed in the trachea, the tip of the bougie is felt clicking as it passes over the tracheal rings [9,10]. When used to exchange out an ETT, the bougie is inserted in the ETT and advanced slowly. The "hold-up sign" is often used as a guide [10]. Once this resistance is encountered, one should not apply additional pressure as trauma to the airway can occur. Complications from the use of a bougie are infrequent but possible. In general, 0.5 to 7% of adverse events associated with difficult intubation are due to trauma [10]. Those from bougie use are even less and typically involve single-use bougies as they are less flexible and the coating makes sliding the ETT over it more difficult [5,11]. The use of a lubricant on the bougie can assist with the latter issue. Because of the tip design (angled instead of straight), there is supposed to be less risk of injury as it is supposed to limit the depth of insertion [11]. Recommended bougie insertion depth has varied from 25cm to 35cm according to some reports, while others used resistance as their guide [12]. Nevertheless, too much pressure, not stabilizing the bougie, or not withdrawing it a couple of cm prior to ETT placement can lead to injury [11]. Maintaining direct visualization (via direct laryngoscopy or Glidescope) during placement of the ETT over the bougie can also help prevent injury [9]. In addition, knowing the length of the patient's airway (determined by height, external anatomy, and imaging studies) may help ensure that the bougie is not placed too deeply [12]. Unfortunately, this information may not be readily available. Damage can occur to any surrounding structures including the larynx, trachea, or branches of the airway [11,13]. There were two reported cases of bougie-associated injury to the upper airway. One report discussed a case of subcutaneous emphysema with a fistula line on the lateral wall following the cricoid cartilage [11], while the other noted creation of a false tissue passage in the trachea leading to complete airway obstruction [13]. In addition, perforation of the trachea or bronchi can further lead to the development of a pneumothorax, which can be potentially detrimental. This was evidenced by several reported cases [2-5,14], all of which involved difficult intubations requiring bougie assistance.

Pneumothorax occurs when air and/or gas enters the space between the lungs and the chest wall causing lung collapse [15-18]. It can develop spontaneously or secondary to trauma. Spontaneous pneumothorax is usually the result of underlying diseases like infections, including tuberculosis or pneumonia, inflammation, and malignancy, or it can occur for unknown reasons [15-18]. Traumatic pneumothorax can be the result of a procedure or intervention (iatrogenic) or due to blunt force or penetrating trauma (non-iatrogenic) [15-16,18]. Procedures more commonly associated with pneumothorax include central line placement, thoracentesis, and mechanical ventilation [2]. Pneumothorax is also an infrequent complication of intubation. This has been seen in association with difficult intubations or the usage of double-lumen tubes [2].

The mechanism by which pneumothorax occurs is often unknown. Potential causes include rupture of pulmonary bullae or blebs, tracheobronchial injury along the posterior wall, tube mispositioning leading to trauma of the lung apex, or torn perivascular alveoli causing air entry via the perivascular sheath of the pulmonary artery [15-18]. High positive-pressure ventilation during mechanical ventilation or high pressures from CPAP usage can also lead to barotrauma and inadvertent pneumothorax, particularly in patients with known lung disease [15-18]. Bronchoscopy or CT scan can be used to determine the mechanism by which pneumothorax occurs [2].

In one reported case, tracheobronchial injury of the posterior wall was thought to be the culprit for pneumothorax [3]. They found that placement of the ETT obscured the tear initially, and once the tube was removed, the injury became evident, and pneumothorax ensued. According to that report, complications from a posterior wall injury can appear as late as 12 hours after the initial injury [3]. In that respect, a posterior wall tear could be a potential cause of pneumothorax in the current case given the delay in presentation. Signs of pneumothorax following intubation typically include increased airway pressure, decreased O_2_ saturation, and hemodynamic instability, usually hypotension [2,15]. On exam, there are little to no breath sounds heard on the affected side [15-18]. In the current case, there were no signs of pneumothorax throughout the procedure. The airway pressures remained within normal limits, the O_2_ saturation was >95% throughout the case, and vital signs were stable. If a posterior wall tear was present, then the injury was likely blocked by the ETT during the procedure. Once the ETT was removed, air would have slowly leaked into the pleural cavity causing a pneumothorax which worsened over time. Other potential causes include a tracheobronchial mucosal tear or perivascular alveolar rupture. In the reviewed case reports, both of these types of injuries led to immediate symptoms requiring treatment [2,4,5]. Therefore, they would not explain the delay in clinical presentation for the current case. 

In the early stage of postoperative recovery, the patient did have decreased oxygen saturation, but this improved with oxygen therapy and nebulizer treatment. The patient was also placed on CPAP because of his history of OSA. Unfortunately, he was unable to tolerate the CPAP and nasal cannula oxygen was used instead. Perhaps, this decreased O_2_ saturation in the PACU and initial inability to tolerate CPAP were early signs that a small pneumothorax was present. A smaller pneumothorax may present with little or no symptoms and often require no treatment [17,18]. A larger pneumothorax, on the other hand, may present with dyspnea, pleuritic chest pain, increased oxygen requirements as well as anxiety, increased work of breathing, cyanosis, increased heart rate, and hypotension [17,18]. If untreated, this can lead to hemodynamic instability, shock, and cardiovascular collapse [16-18]. Because the diagnosis is primarily clinical, a high level of suspicion is necessary when the above symptoms develop, especially following a procedure or difficult intubation. Diagnosis is generally confirmed with CXR, but CT scan and Ultrasound may also be used [18]. Conservative therapy may be appropriate for a smaller pneumothorax, but larger ones require treatment with a chest tube to help re-inflate the lung [16-18]. Typically, there is immediate improvement in breathing and oxygen saturation after tube thoracostomy [16-18]. The chest tube is kept in place for several days before it is removed. Recovery is usually uneventful, and there are typically no long-term complications.

## Conclusions

Nasal intubation is a common technique during general anesthesia, and the bougie is often used to guide the ETT into the trachea. Bougies are generally safe if used appropriately; however, complications can occur. When using a bougie to assist with intubation, care must be taken not to apply too much pressure or force on insertion into the trachea, especially when resistance is encountered. If intubation is challenging, the patient must be monitored closely in the entirety of the perioperative period as problems may not be immediate. Pneumothorax is an infrequent but significant complication associated with intubation and has been linked to bougie usage. The diagnosis is primarily clinical, emphasizing the need for heightened awareness by providers. With persistent monitoring, signs and symptoms will not be overlooked, and the patient can receive an accurate diagnosis and appropriate treatment. This was a case of a delayed pneumothorax following a difficult intubation. Even when precautions are taken and preparations are made, a seemingly routine procedure can lead to a clinically significant adverse event. Cognizance of such potential complications is crucial for optimizing patient safety during airway management procedures and calls for a careful reassessment of the risks associated with using a bougie.
